# Genome Sequence of Human Cytomegalovirus Ig-KG-H2, a Variant of Strain KG Propagated in the Presence of Neutralizing Antibodies

**DOI:** 10.1128/MRA.00063-20

**Published:** 2020-04-23

**Authors:** Ahmed Al Qaffas, Salvatore Camiolo, Jenna Nichols, Andrew J. Davison, Amine Ourahmane, Xiaohong Cui, Mark R. Schleiss, Laura Hertel, Dirk P. Dittmer, Michael A. McVoy

**Affiliations:** a Department of Pediatrics, Virginia Commonwealth University, Richmond, Virginia, USA; b MRC-University of Glasgow Centre for Virus Research, Glasgow, United Kingdom; c Center for Infectious Diseases and Microbiology Translational Research, Division of Pediatric Infectious Diseases, Minneapolis, Minnesota, USA; d Department of Pediatrics, School of Medicine, University of California, San Francisco, Oakland, California, USA; e Lineberger Comprehensive Cancer Center Program in Global Oncology, Department of Microbiology and Immunology, Center for AIDS Research, School of Medicine, University of North Carolina at Chapel Hill, Chapel Hill, North Carolina, USA; KU Leuven

## Abstract

Human cytomegalovirus shed in infant urine was isolated and serially passaged in fibroblasts in the presence or absence of neutralizing antibodies. Comparison of the genome sequences of representative viruses Ig-KG-H2 (passed with antibody) and ϕ-KG-B5 (passed without antibody) revealed the presence of several mutations in each virus.

## ANNOUNCEMENT

Human cytomegalovirus (HCMV) replicates poorly when first isolated from clinical material. However, upon serial passage in fibroblasts, improved replication and increased release of cell-free virus are conferred by disruptive mutations in the *RL13* gene and one or more of the contiguous genes *UL128*, *UL130*, and *UL131A* (1). The latter mutations disrupt assembly of a pentameric complex on the virion surface that is important for entry into epithelial and endothelial cells but not fibroblasts (2–6).

In our recent work, replicate fibroblast cultures were infected with HCMV in urine from a symptomatic congenitally infected infant (7). One lineage (Ig-KG) was passaged with HCMV-hyperimmune globulin (HIG) (CytoGam) in the culture medium, whereas the other (ϕ-KG) was passaged in the absence of HIG. ϕ-KG lost epithelial tropism and acquired frameshift mutations disrupting *RL13* and *UL131A*, whereas Ig-KG retained epithelial tropism and was intact in these genes after 22 passages. Long-term genetic stability of these lineages (and their mutations) was confirmed by isolating representative viruses by limiting dilution, i.e., Ig-KG-H2 from the Ig-KG passage 22 stock and ϕ-KG-B5 from the ϕ-KG passage 13 stock.

Preliminary Ion Torrent and targeted PCR/Sanger sequence analyses that were focused on protein-coding sequences identified mutations affecting five genes (*RL13*, *UL100*, *UL102*, *UL122*, and *UL131A*) in the parental Ig-KG and ϕ-KG stocks that were also present in Ig-KG-H2 and ϕ-KG-B5 (7). Here, we report the complete genome sequence of Ig-KG-H2 and compare it with that of ϕ-KG-B5.

Ig-KG-H2 or ϕ-KG-B5 virions were pelleted from culture supernatants by ultracentrifugation and treated with DNase I prior to DNA purification by proteinase K digestion, phenol-chloroform extraction, and ethanol precipitation (7). DNA samples (∼100 ng) were sheared acoustically to an approximate size of 450 bp, and sequencing libraries were processed through seven PCR cycles using the LTP library preparation kit (KAPA Biosystems, Wilmington, MA, USA), employing NEBNext multiplex oligos for Illumina (New England Biolabs, Ipswich, MA, USA). Sequencing on an Illumina NextSeq mid-output 300-cycle cartridge generated 9,614,942 and 10,011,260 paired-end reads of 150 nucleotides for Ig-KG-H2 and ϕ-KG-B5, respectively. A pipeline included in the GRACy tool (https://github.com/salvocamiolo/GRACy) was used to perform *de novo* assembly of the Ig-KG-H2 reads. Briefly, reads that aligned with the Hg38 human reference sequence (GenBank GCA_000001405.15) using Bowtie 2 v. 2.3.1 (8) (with the end-to-end flag set) were removed, and sequencing adapters and low-quality reads were removed using Trim Galore v. 0.4.0 (https://github.com/FelixKrueger/TrimGalore) and PRINSEQ v. 0.20.4 (9), respectively. The remaining reads were normalized and assembled using SPAdes v. 3.12 (10), and the resulting contigs were ordered in relation to the HCMV reference strain Merlin genome sequence (GenBank accession number AY446894.2). Gaps were closed using an overlap-layout-consensus algorithm implemented in GRACy, and the assembly was further refined by visualization in Tablet v. 1.19.09.03 (11) of a read alignment that had been generated using Bowtie 2. All tools were used with default parameters unless otherwise specified. The Ig-KG-H2 genome sequence consisted of 236,244 bp (G+C content, 57.4%) and was determined at an average coverage of 4,886 reads/nucleotide. The ϕ-KG-B5 reads were aligned to the resulting Ig-KG-H2 genome sequence using Bowtie 2, and differences present in the entire population were identified manually using Tablet.

As reported previously (7), ϕ-KG-B5 had disruptive mutations in *RL13* and *UL131A*, as well as a single amino acid substitution in *UL122* (Table 1). In contrast, Ig-KG-H2 lacked disruptive mutations in *RL13* and *UL128*, *UL130*, or *UL131A* but contained mutations resulting in four amino acid substitutions in *UL100*, two amino acid substitutions in *UL102*, and a distinct single amino acid substitution in *UL122*. Also, Ig-KG-H2 had two silent mutations in *UL57* and *UL98*. The availability of the genome sequences of Ig-KG-H2 and ϕ-KG-B5 will facilitate studies of the relative importance of these mutations in the adaptation of Ig-KG-H2 to growth in the presence of HIG.

**TABLE 1:** Mutations identified in the Ig-KG-H2 and ϕ-KG-B5 genomes

Gene	Protein	Mutant^*a*^	Mutation(s)^*b*^	Consequence
None	None	ϕ-KG-B5^*c*^	1-bp deletion (C6372)	None
*RL13*	Membrane protein RL13	ϕ-KG-B5	10-bp deletion (CATTATTATT at positions 11661–11670)	Frameshift after residue 164
*UL57*	Single-stranded DNA-binding protein	Ig-KG-H2	C89864T substitution	Silent
*UL98*	DNase	Ig-KG-H2	C145699T substitution	Silent
*UL100*	Envelope glycoprotein M	Ig-KG-H2	C146566G substitution	E361D
			C146750A and T146751G substitutions	S300L
			C146794A substitution	Q286H
			C147608A substitution	S15I
*UL102*	Helicase-primase subunit	Ig-KG-H2	C147895G substitution	L23V
			C148861G substitution	L345V
			C149640T substitution	Silent
*UL122*	Regulatory protein IE2	ϕ-KG-B5	G171290C substitution	F384L
		Ig-KG-H2	G171315T substitution	S376Y
*UL131A*	Envelope protein UL131A	ϕ-KG-B5	1-bp insertion (T178079)	Frameshift after residue 27

a The virus in which each mutation occurred was identified by comparison with strain Merlin as a representative HCMV strain.

b Coordinates refer to the Ig-KG-H2 genome sequence.

c This is only nominally a mutant, as the mutation represents a difference in the number of nucleotides in a C tract that varies in length among HCMV strains.

### Data availability.

The genome sequence of Ig-KG-H2 has been deposited in GenBank under accession number MN274568. Raw reads are available from the European Nucleotide Archive with accession numbers ERR3988552 (Ig-KG-H2) and ERR3988553 (ϕ-KG-B5).
